# Proteomic profile and morphological characteristics of skeletal muscle from the fast- and slow-growing yellow perch (*Perca flavescens*)

**DOI:** 10.1038/s41598-021-95817-7

**Published:** 2021-08-11

**Authors:** Karolina Kwasek, Young Min Choi, Hanping Wang, Kichoon Lee, John Mark Reddish, Macdonald Wick

**Affiliations:** 1 Center for Fisheries, Aquaculture, and Aquatic Sciences, School of Biological Sciences, Southern Illinois University, Carbondale, IL USA; 2 Department of Animal Science and Biotechnology, Kyungpook National University, Sangju, South Korea; 3 Ohio Center for Aquaculture Research and Development, The Ohio State University South Center, Piketon, OH USA; 4 Department of Animal Sciences, The Ohio State University, Columbus, OH USA; 5 School of Veterinary Medicine, The Ohio State University, Columbus, OH USA

**Keywords:** Physiology, Zoology, Proteomics

## Abstract

The objective of the present study was to compare skeletal muscle proteomic profiles, histochemical characteristics, and expression levels of myogenic regulatory factors (MRFs) between fast- versus slow-growing yellow perch *Perca flavescens* and identify the proteins/peptides that might play a crucial role in the muscle growth dynamic. Yellow perch were nursed in ponds for 6 weeks from larval stage and cultured in two meter diameter tanks thereafter. The fingerlings were graded to select the top 10% and bottom 10% fish which represented fast- and slow-growing groups (31 yellow perch per each group). Our statistical analyses showed 18 proteins that had different staining intensities between fast- and slow-growing yellow perch. From those proteins 10 showed higher expression in slow-growers, and 8 demonstrated higher expression in fast-growers. Fast-growing yellow perch with a greater body weight was influenced by both the muscle fiber hypertrophy and mosaic hyperplasia compared to slow-growing fish. These hyperplastic and hypertrophic growth in fast-grower were associated with not only metabolic enzymes, including creatine kinase, glycogen phosphorylase, and aldolase, but also myoD and myogenin as MRFs. Overall, the results of the present study contribute to the identification of different expression patterns of gene products in fast- and slow-growing fish associated with their muscle growth.

## Introduction

The goal of the aquaculture industry is to intensify fish production and enforce fish growth. Fish growth is mostly reflected by muscle growth, and more specifically the white muscle development. This process in most fish species is unique compared to, for example, birds or mammals, since it is determined by not only hypertrophy—the growth of existing muscle fibers, but also by hyperplasia—fiber recruitment, which continues throughout the fish life^1^. This constant recruitment of muscle fibers seems essential for the fish in order to achieve larger size, and fiber development and growth have been linked to increased expression of myogenic regulatory factors (MRFs) known for encoding proteins that regulate myogenesis and muscle growth^1^. The white twitch muscle fibers are the main edible part of the fish muscle, hence, understanding the interactions between environmental and genetic factors that affect muscle cellularity (fiber number, size, etc.) as well as expression of muscle building blocks—proteins, is crucial for producing fish of desired size and quality.

The proteins expressed in skeletal muscle play important role in various biological processes. Some of those proteins have been identified to be associated with body weight and length of yellow perch *Perca flavescens* and included enzymes involved in glycolytic pathway or process of muscle contraction^2^. Some of the myofibrillar proteins that affect fish muscle growth include myosin heavy chain (MHC), which has been shown a differentiated expression under various environmental conditions^3^. It was reported, for example, that the MHC mRNA level decreased when insufficient amount of protein was provided in a diet, and consequently, the synthesis of that myofibrillar protein was also reduced^4^. Additionally, expression of sarcoplasmic proteins has been associated with different metabolic pathways; thus, patterns of these proteins can provide some insight into the metabolic status related to muscle growth^5^. However, expression patterns of these proteins associated with muscle growth in yellow perch are largely unknown.

Fish muscle studies are of major economic importance since the flexibility of muscle growth under different conditions determines the muscle mass and meat quality^6^. Selection for faster growth has potential to contribute to not only greater feed consumption and more efficient feed utilization, but also higher production yields and shorter production cycles^7^. Therefore, the specific traits used for selection of individuals for faster muscle growth need to be established. More importantly, the molecular mechanisms, including regulation of skeletal muscle protein expression underlying faster growth need to be identified. Moreover, muscle growth attributes should be assessed whether it is growth by fiber hyperplasia or fiber hypertrophy by histochemical and genetic methods, as these characteristics have a significant impact on growth rate and ultimate muscle mass^8^. Hence, the objectives of the present study were to compare skeletal muscle proteomic profiles of fast- versus slow-growing yellow perch and identify the proteins/peptides that might play a crucial role in muscle growth dynamic. Additionally, this study compared muscle fiber characteristics and MRFs expression levels between fast- and slow-growing yellow perch in order to provide a new model for increased muscle growth in the aquaculture area.

## Materials and methods

### Fish mating, fry production, and grading

The animals were maintained in strict accordance with the recommendations in the Guide for the Care and Use of Laboratory Animals of The Ohio State University (OSU). The OSU Institutional Animal Care and Use approved all of the protocols performed (protocol# 2012A00000133-R2). Animal study was carried out in compliance with the ARRIVE guidelines (https://arriveguidelines.org). All researchers were trained in accordance with OSU Institutional Animal Care and Use requirements. Twenty pairs of broodfish with passive integrated transponder (PIT) tags were selected from 2006 year-class and 2007 year-class broodstock from the Ohio Genetic Improvement of Farmed-fish Traits (O’GIFT) Program in the Ohio State University (OSU) South Centers, and 20 pair-matings of the two year-classes were made in spring^9^. For each pair-mating, 1 female and 1 male were placed in a 55 L round tank for spawning. Fertilized eggs were incubated in 25 L round tanks with flow-through well water for 11–12 d at a temperature of 11–12 °C. Similar numbers of fry from 20 mating-sets were stocked into a 0.1 ha pond for nursery. The fish were nursed in the pond by the pond-fertilization method for 6 weeks. Subsequently, feed training was conducted in 400 L round tanks for 3 weeks^10^. After the feed training, fish were reared in a two meter diameter tank for two months. Then top 10% and bottom 10% fish were graded by length with bar grader, respectively, to obtain the fast and slow growing fish for this study. Commercial floating feed (Silver Cup, 45% protein, 16% fat; Nelson and Sons Inc., Murray, UT) was used during the periods of feed training and tank rearing.

Two hundred-eleven fast-growing fish of mean size 16.4 ± 6.8 g and 184 slow-growing fish of mean size 3.3 ± 1.1 g were randomly selected from fast- and slow-growing populations and immediately immersed in liquid nitrogen. Samples were then transferred to − 80 °C. Thirty-one fish were then randomly selected from each group for proteomic, histochemical, and western blot analyses. The mean size of those fish was 16.4 ± 5.7 g and 3.3 ± 0.6 g for fast- and slow-growing groups, respectively.

### Proteomic analysis

Fish muscle proteins were extracted as previously described in Zapata et al.^11^. Briefly, 50 mg of muscle samples were homogenized in 1 mL of low-salt buffer (50 mM NaCl, 0.1% NaN3 0.4 mM Pefabloc SC Plus (Boehringer Mannheim Corp., Indianapolis, IN, USA) and the soluble sarcoplasmic protein fraction was separated from the insoluble myofibrillar protein fraction by centrifugation at 10,000×*g* at 4 °C for 5 min. Samples (500 µl) of the sarcoplasmic fraction and 60 mg of the myofibrillar fraction were solubilized in 8 M urea, 2 M thiourea, 3% SDS, 50 mM Tris, 0.004% bromophenol blue, 75 mM DTT solution at a ratio of 1:2 and incubated at room temperature for 1 h. Samples were centrifuged for 5 min at 10,000×*g* at room temperature prior the electrophoretic analysis. Samples were then loaded onto 1 mm × 12 cm × 14 cm 10% polyacrylamide gel with 3% stacking gel. Gels were run at a constant voltage of 10 cm^−1^ until the dye front ran off from the bottom of the gel. After electrophoretic separation gels were stained overnight in Sypro Ruby protein stain following manufacturer’s protocol (BioRad Laboratories, Hercules, CA, USA). The gels were scanned on a Typhoon 9400 laser scanner (GE healthcare, Chalfont St., Giles, UK)^11^. Digital images were analyzed by using Total-Lab TL120 software (Nonlinear Dynamics Inc., Durham, NC, USA). The bands were analyzed to determine the percentage contribution of each band to the total band area in the lane. The following parameters in the software were used: background subtraction: rolling ball with radius = 300; band detection: minimum slope = 30, noise reduction = 3, % maximum peak = 1; profile deconvolution: Gaussian volumes fitted to peaks = no advanced fitting; Rf calibration aligned with no lane, and use curve lines were checked. Bands that were detectable between 21 and 223 kDa were selected for statistical analysis.

Bands that were identified as significant were excised from gels and analyzed by mass spectrometry at the Campus Chemical Instrument Center MS and Proteomics Facility of The Ohio State University. Gels were digested with trypsin from Promega (Madison, WI, USA) using the Montage In-Gel Digestion Kit from Millipore (Bedford, MA, USA) following the manufacture’s protocols. Briefly, bands were trimmed and washed in 50% methanol/5% acetic acid for 1 h. The gel bands were then incubated with a DTT solution (5 mg/mL in 100 mM ammonium bicarbonate) for 30 min. A 15 mg/mL of iodoacetamide in 100 mM ammonium bicarbonate solution was the added. The gel bands were incubated in the dark for 30 min before their removal. The gel bands were then washed with cycles of ACN and ammonium bicarbonate (100 mM) in 5 min increments. The gel bands were dried in a speed vac and then protease was driven into the gel pieces by rehydrating them in 50 mL of sequencing grade modified trypsin at 20 mg/mL in 50 mM ammonium bicarbonate for 10 min. Twenty micro-liters of 50 mM ammonium bicarbonate were added to the gel fragments and the mixture was incubated at room temperature. The peptides were extracted from the gel with 50% ACN and 5% formic acid. The extracted pools were concentrated to 25 mL in a speed vacuum. Capillary Nano-LC/MS/MS was performed on a Thermo Finnigan LTQ mass spectrometer equipped with a nanospray source operated in positive ion mode. The LC system was an UltiMate Plus system from LC-Packings A Dionex (Sunnyvale,CA, USA) with a Famos auto sampler and Switchos column switcher. Solvent A contained water with 50 mM acetic acid and solvent B contained ACN. A volume of 5 mL of each sample was injected on to the trapping column (LC-Packings A Dionex) and washed with 50 mM acetic acid. The injector port was switched to inject and the peptides were eluted off the trap onto the column. A 5 cm, 75 mm I-D ProteoPep II C18 column (New Objective, Woburn, MA, USA) packed directly in the nanospray tip was used for chromatographic separations. Peptides were eluted directly from the column into the LTQ system A gradient of 2–80% B over 50 min, with a flow rate of 300 nL/min was used. The MS/MS was acquired according to standard conditions established at the Campus Chemical Instrument Center MS and Proteomics Facility of The Ohio State University. Briefly, a nanospray source operated with a spray voltage of 3 kV was used. The temperature of the capillary was 200 °C. The Top Ten™ method for scan sequence of the mass spectrometer was used. The analysis was programmed for a scan recorded between 350 and 2000 Da, and a MS/MS scan to generate product ion spectra to determine amino acid sequence inconsecutive instrument scans of the ten most abundant peaks in the spectrum. The CID fragmentation energy was set to 35%. Dynamic exclusion was enabled with a repeat count of 30 s, exclusion duration of 350 s and a low mass width of 0.5 Da and high mass width of 1.50 Da. The sequence data from the MS/MS was processed by transforming the data files into a merged file (.mgf) using MGF creator (merge.pl, a Perl script). The mgf files were then searched using MASCOT Daemon by Matrix Science (Boston, MA, USA). Data processing was performed following the guidelines described by Wilkins et al.^12^. Assigned peaks had a minimum of ten counts. Protein identifications were analyzed manually and proteins with a minimum of two unique peptides from one protein or more were accepted. The abundance of identified proteins was estimated by calculating the exponentially modified protein abundance index (emPAI) according to Ishihama et al.^13^.

### Muscle fiber characteristics

White muscle samples were taken from near the dorsal fin on the left side of the fish^8^, and frozen in liquid nitrogen to minimize muscle fiber damage, and then stored at − 80 °C until the analysis of muscle fiber characteristics could be performed. Serial transverse muscle sections (10 μm) were obtained from each sample using a cryostat (CM1850, Leica Microsystems, Wetzlar, Germany) at − 25 °C, and where then mounted onto glass slides. The myosin ATPase activity of the samples was detected following pre-incubation with acid (4.7) or alkaline (10.7) pH^14^. All muscle fibers of yellow perch in this study were identified as fast-twitch glycolytic fiber (type II fiber). Additionally, the hematoxylin and eosin (H&E) staining method were performed to evaluate the histochemical characteristics^15^. All histochemical samples from the H&E staining method were examined using an optical microscope equipped with a CCD color camera (IK-642K, Toshiba, Tokyo, Japan) and a standard workstation computer that controlled the image analysis system (Image-Pro Plus, Media Cybernetics, Silver Spring, LP, USA). All portions of the sections analyzed were carefully handled to avoid tissue disruption and freeze-damage. At least 300 fibers were evaluated per sample. The average cross sectional area (CSA) of the muscle fibers was determined as the ratio of the total area measured divided by the total number of fibers. The distribution of fiber CSA was calculated as the percentage of the total fiber number of each CSA sections (0 to 300, 301 to 600, 601 to 900, 901 to 1200, 1201 to 1500, 1501 to 1800, 1801 to 2100, 2101 to 2400, 2401 to 2700, 2701 to 3000, and more than 3001) to the total fiber number.

### Protein isolation and western blot analysis

Muscle samples were homogenized in ice-cold 1 X lysis buffer (62.5 mM Tris, 5% SDS) with a Tissuemiser (Fisher Scientific, Pittsburgh, PA, USA) and combined with 2 X Laemmli buffer (Bio-Rad Laboratories, Hercules, CA, USA) containing 62.5 mM Tris, 1% SDS, 5% 2-mercaptoethanol, 12.5% glycerol, and 0.05% bromophenol blue^16^. Western blot analysis was performed by a previously reported method^17^. Proteins were separated by SDS-PAGE and wet-transferred to a 0.45 µm pore polyvinylidene fluoride membrane (Millipore, Billerica, MA, USA). Membranes were blocked in 5% nonfat dry milk in tris-buffered saline-tween (TBST; 20 mM Tris, 150 mM NaCl, pH 7.4, plus 0.15% Tween 20) for 1 h at room temperature. Membranes were incubated overnight at 4 °C in primary antibodies: myogenin (1:1000 Santa Cruz Biotechnology Inc., Santa Cruz, CA, USA), myoD (1:2500 Santa Cruz Biotechnology Inc.), and β-actin (1:2000 Santa Cruz Biotechnology Inc.). Membranes were then washed in TBST for 1 h and incubated with horseradish peroxidase-conjugated secondary antibodies (Santa Cruz Biotechnology Inc.) for 2 h at room temperature. Membranes were washed again for 1 h in TBST followed by detection with ECL Plus (GE Biosciences, Piscataway, NJ, USA). The membranes were exposed to Bio-Max X-ray film (GE Biosciences) for visualization of the myogenin, β-actin, and myoD proteins. For the purpose of quantitative analysis, each protein band was examined by an image analysis system (Kodak 1-D Image Analysis Software, Eastman Kodak Company, Rochester, NY, USA). MyoD/β-actin was determined as the ratio of the myoD band intensity divided by the β-actin band intensity, and myogenin/β-actin was determined as the ratio of the myogenin band intensity divided by the β-actin band intensity. The intensities of myoD and myogenin bands were normalized by band intensities of the slow-growing group.

### Statistical analyses

Regarding the characteristics of muscle fiber and expression levels of MRFs, the general linear model procedure was performed for the association between the fast-and slow growing fish groups using SAS (SAS institute, Cary, NC, USA). The staining intensities of identified gel bands between fast- and slow-growing yellow perch were analyzed using *t*-test by SPSS version 17.0 (SPSS Inc., Chicago, IL). The nonparametric Mann–Whitney test was used to compare differences between the two independent groups when abnormal distribution was detected. Significant differences between groups were detected by the probability difference, and were considered significant at p < 0.05.

## Results

### Proteomic analysis

**Figure 1:**
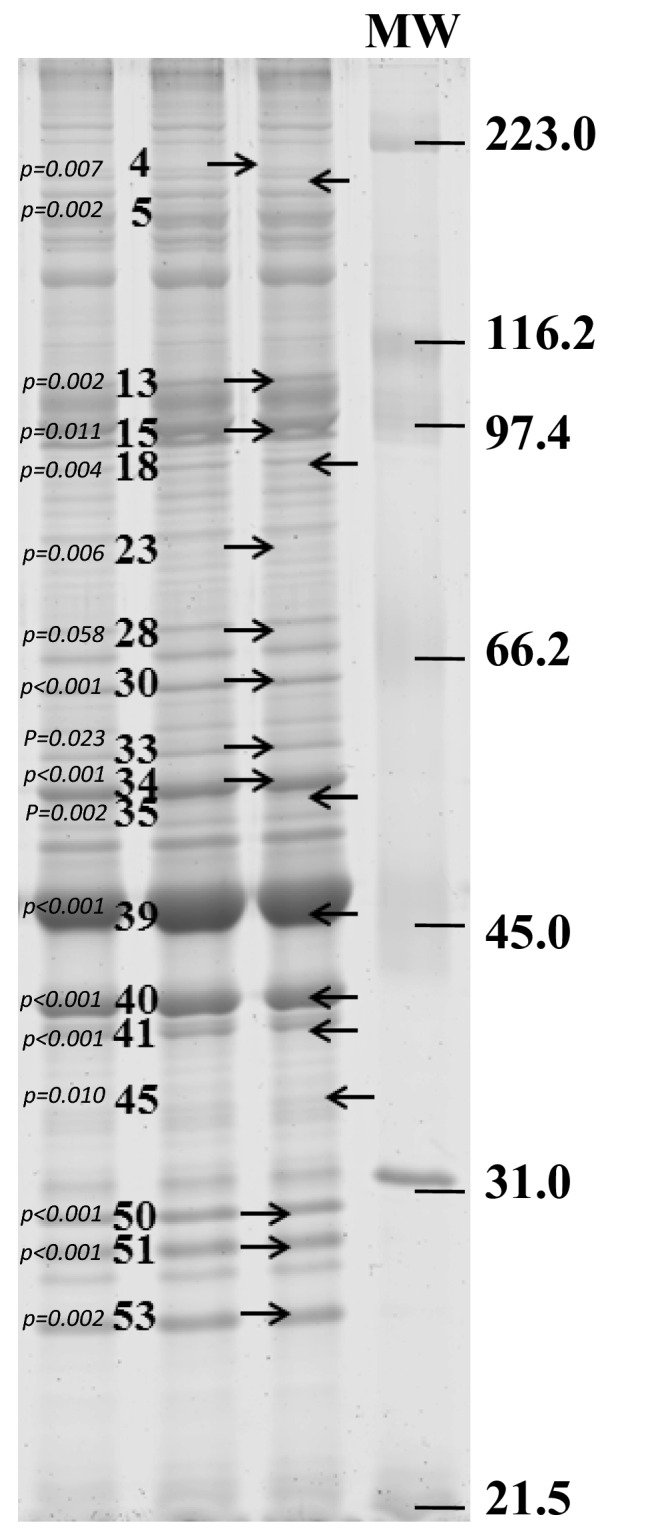
Representative lanes from a 10% SDS-PAGE of the sarcoplasmic fraction of the muscle of fast- and slow-growing yellow perch. The bands were analyzed to determine the percentage contribution of each band to the total band area in the lane. The arrows indicate bands that were different between groups. The marker is the molecular weight standard. Figure presents cropped gel.

The electrophoretic analyses identified 58 bands out of which 18 differed in staining intensity between fast- and slow-growing yellow perch. Figure 1 presents representative lanes of the sarcoplasmic fraction of yellow perch muscle showing regions of the gel in which bands between both groups, slow- and fast-growing fish, had different staining intensities. The 18 bands that were different, labeled by arrows, were band numbers: 4, 5, 13, 15, 18, 23, 28, 30, 33, 34, 35, 39, 40, 41, 45, 50, 51, and 53.

Ten bands showed higher staining intensity in slow-growers, while eight other bands showed higher staining intensity in fast-growers. Table 1 presents sequencing data of bands that were found different between fast- and slow-growing yellow perch and their emPAI values. Again, the Mowse Score was calculated using MASCOT. Mass prediction is from protein identification in NCBI BLAST protein database. Nine bands out of 18 that differed between groups were identified. In fast-growing fish the following proteins were found: actinin alpha 3a, glycogen phosphorylase, phosphoglucose isomμerase-2, F1 ATP synthase beta unit, enolase 1, beta-enolase, creatine kinase, aldolase A, glyceraldehyde 3-phosphate dehydrogenase (GAPDH), lactate dehydrogenase, and adenylate kinase isoenzyme 1. In slow-growing fish: alpha actinin, sarcoendoplasmic reticulum calcium ATPase, phosphoglucose isomerase 2, creatine kinase, and GAPDH were recognized. All of these proteins except for actinin alpha 3a and phosphoglucose isomerase 2, were found in bands which had higher staining intensities in the fast-growing group of fish.

**Table 1:** Proteins from 10% SDS-PAGE gel which were associated with bands with different staining intensities between fast- and slow-growing yellow perch.

Accession no. (gi)	Band no	Protein ID	Species	Mass^a^	Mowse score^b^	Peptide matches	Sequences	emPAI^c^
**Fast-growing yellow perch**								
56118264	13	Actinin alpha 3a	*Danio rerio*	104072	404	12	6	0.2
66472494	15	Glycogen phosphorylase muscle form	*Danio rerio*	97384	555	16	8	0.35
20067651	30	Phosphoglucose isomerase-2	*Mugil cephalus*	621652	479	11	5	0.43
226441961	33	F1 ATP synthase beta subunit	*Gillichthys seta*	53949	505	13	6	0.51
37590349	33	Enolase 1, (alpha)	*Danio rerio*	47386	297	6	3	0.22
47551317	34	beta-enolase	*Danio rerio*	47841	553	18	7	0.7
31322099	39	Creatine kinase muscle isoform 2	*Chaenocephalus aceratus*	42884	885	37	10	1.83
22671688	39	Aldolase A	*Danio rerio*	40214	195	10	2	0.27
25989185	40	Glyceraldehyde 3-phosphate dehydrogenase	*Gadus morhua*	36241	621	23	7	1.02
17433107	41	Lactate dehydrogenase chain A	*Sphyraena idiastes*	36547	230	7	3	0.3
25989185	41	Glyceraldehyde 3-phosphate dehydrogenase	*Gadus morhua*	36241	188	3	3	0.3
308322207	53	Adenylate kinase isoenzyme 1	*Ictalurus furcatus*	21588	392	13	6	1.76
**Slow-growing yellow perch**								
25992501	13	Alpha-actinin	*Danio rerio*	104478	453	9	6	0.2
110750739	13	Sarcoendoplasmic reticulum calcium ATPase	*Silurus lanzhouensis*	110237	304	8	4	0.12
20067651	30	Phosphoglucose isomerase 2	*Mugil cephalus*	62165	180	3	2	0.11
31322099	39	Creatine kinase muscle isoform 2	*Chaenocephalus aceratus*	42884	481	11	5	0.45
308321716	40	Glyceraldehyde-3-phosphate dehydrogenase	*Ictalurus furcatus*	36037	252	5	3	0.3

^a^Mass predicted from protein identification in NCBI BLAST protein database.

^b^Score calculated using MASCOT.

^c^Exponentially modified protein abundance index.

### Muscle fiber characteristics

**Figure 2:**
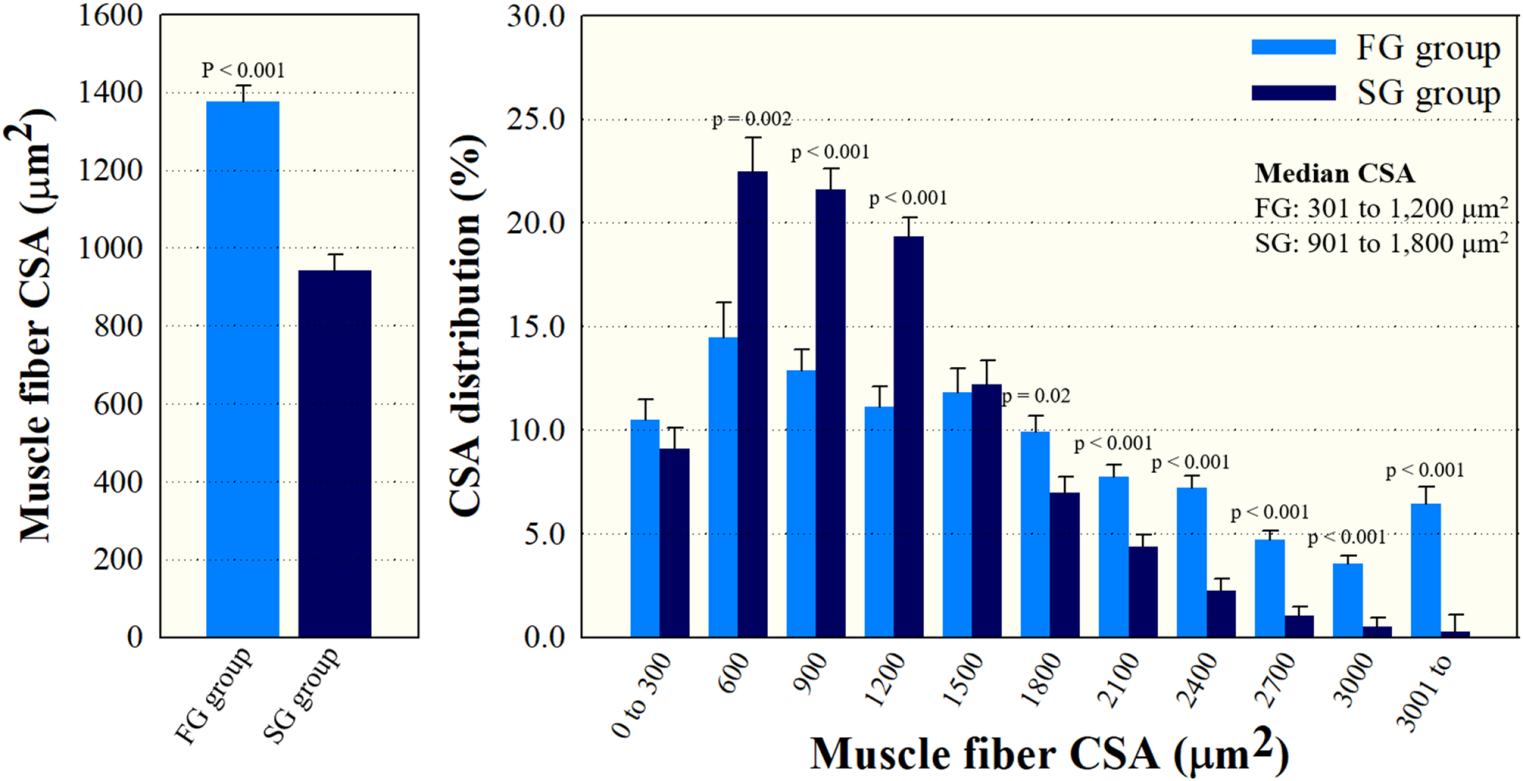
Mean cross-sectional area (CSA) and CSA composition of muscle fiber of fast- (FG) and slow-growing (SG) yellow perch. Results were represented as means and SEM shown by vertical bars.

**Figure 3:**
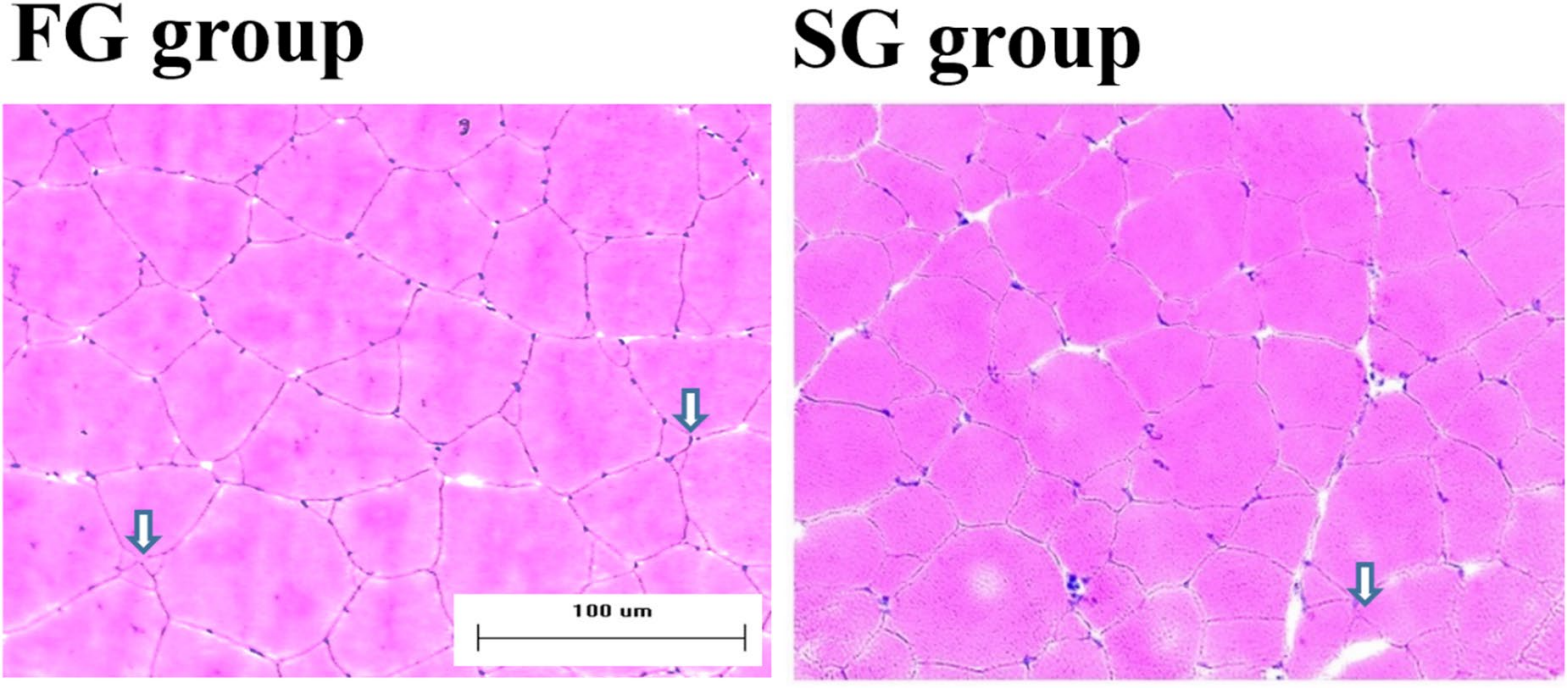
Morphological characteristics of muscle fiber in fast-growing (FG) and slow-growing (SG) yellow perch. Mosaic hyperplasia of fiber was often observed in fast-growing yellow perch, and was represented by white arrow.

Figure 2 presents the morphological characteristics of muscle fiber, including fiber CSA and CSA composition, in yellow perch categorized by body weight. Marked differences were detected in muscle fiber CSA between the groups (p < 0.001), and fast-growing yellow perch exhibited a greater area compared to slow-growing yellow perch (1377 vs. 942 μm^2^). As expected, slow-growing fish showing a lower body weight showed higher percentage of smaller fibers (301 to 600, 601 to 900, and 901 to1200 μm^2^; p < 0.05), even though no significant difference between the groups was observed in the smallest fiber (0 to 300 μm^2^; p = 0.33). The percentage of 1800 to 2100, 2101 to 2400, 2401 to 2700, and 2701 to 3000 fibers was significantly higher in fast-growing yellow perch compared to slow-growing yellow perch (p < 0.05). Additionally, a mosaic form of muscle fiber was often observed in fast-yellow perch, and CSA of these fibers was small (< 600 μm^2^; Fig. 3).

### Expression levels of myoD and myogenin

**Figure 4:**
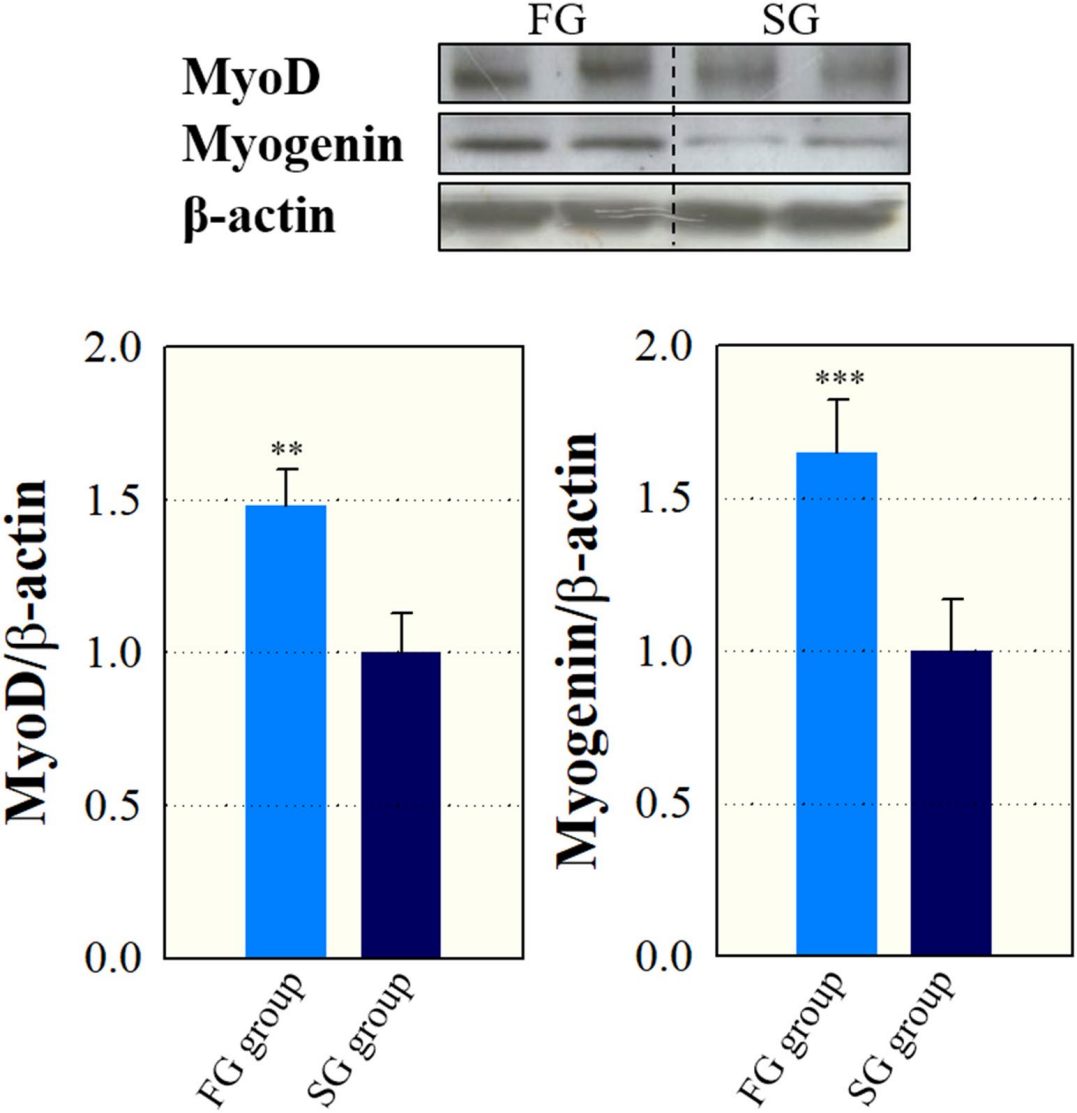
Western blot images and expression of myoD and myogenin in fast- (FG) and slow-growing (SG) yellow perch. For western blot analysis of myoD and myogenin, the expression level of β-actin was used as a control. Results were represented as means and SEM shown by vertical bars.

Expression levels of myogenic markers, including myoD and myogenin, are assessed by immunoblotting analysis, and graphically presented in Fig. 4. The expression levels of myoD (1.48 vs. 1.00, p = 0.01) and myogenin (1.65 vs. 1.00, p < 0.001) were significantly greater in fast-growing yellow perch compared to slow-growing yellow perch.

## Discussion

The aquaculture industry requires an improvement in methods for proper selection of fast-growing fish reflected by an enhanced growth performance of the myotomal muscle—the largest tissue fraction (~ 70%) in majority of fish species^18^. Fish white muscle constitutes of fast-contracting muscle fibers using the glycolytic metabolic pathways in rapid movement (i.e. escape) as opposed to the red muscle fibers used to power slow speed movements. In the present study we compared skeletal muscle sarcoplasmic proteins/peptides mainly associated with metabolic pathways staining intensities between fast- and slow-growing yellow perch in order to identify the differences in expression of skeletal muscle proteins in fish exhibiting different growth capacities by using 1D electrophoresis followed by image and statistical analysis. This study identified 58 bands and 18 of those were characterized by different staining intensities between fast- and slow-growing yellow perch. Nine of those bands identified proteins/peptides and those were predominantly metabolic enzymes, such as: phosphoglucose isomerase 2, enolase (alpha and beta), GAPDH, glycogen phosphorylase (muscle isoform), lactate dehydrogenase chain A, F1 ATP synthase beta subunit, adenylate kinase isoenzyme 1, and creatine kinase muscle isoform 2. All of these proteins with the exception for phosphoglucose isomerase 2 were associated with bands that presented higher staining intensities in fast-growing fish compared to slow-growing fish suggesting possible association of these proteins with faster growth of yellow perch when expressed at higher levels. Nagakawa and Nagayama^19^ reported aldolase to be one of the major sarcoplasmic proteins in muscle of a number of fish species. Aldolase is considered an enzyme of glycolysis since it converts fructose 1,6-bisphosphate into dihydroxyacetone phosphate and glyceraldehydes 3-phosphate, both precursors to glycolysis and gluconeogenesis. Consequently, the higher expression rates of aldolase as well as other enzymes also involved in glycolytic pathways (enolase, phosphoglucose isomerase, GAPDH) might reflect increased ATP turnover required to support the rapid growth. The reduced expression in slow-growers could oppositely reflect a way of saving energy by diminishing production of the same group of proteins^20^. The phosphoglucose isomerase 2 was associated with band 30, which had higher staining intensity in the slow-growing group of fish. However, the emPAI results for this protein showed greater value in the fast-growing group (0.43) compared to the slow-growing group (0.11) indicating higher amounts of this protein in the “fast” group of fish. Therefore, it is possible that the higher staining intensity of that band in slow-growing fish was associated with other unidentified protein in that band.

Holterhoff et al.^21^ reported a higher expression of actinin-alpha 3a in the axial muscles of zebrafish *Danio rerio.* This protein contributes to physiological specialization of muscles and in the present study it was found in band 13 of both fast- and slow-growing fish, which showed higher staining intensity in the slow-growing group. However, the emPAI values for actinin alpha 3a between fast- and slow fish were the same. Since there was another protein, sarcoendoplasmic reticulum calcium ATPase, in that same band (band 13) identified only in the slow-growing fish it is possible that this protein might have been associated with the higher intensity of band 13 in the slow-growing group of fish. On the other hand, creatine kinase was associated with band 39 in both fast and slow groups of fish. The aldolase A was also associated with band 39 but only in the fast-growing group of fish. The emPAI values for creatine kinase and aldolase A in the fast-growing group were 1.83 and 0.27, respectively. In addition, the emPAI value for creatine kinase in the slow-growing group was 0.45 suggesting that higher expression of this particular protein occurred in fish exhibiting faster growth. Creatine kinase belongs to group of enzymes responsible for catalysis of the reversible phosphoryl transfer from phosphocreatine to ADP. There are several creatine kinase isoenzymes present in tissues with high-energy demands, such as, brain, heart, and skeletal muscle^22,23^. It is possible that the downregulation of creatine kinase in slow-growing fish leads to reduction in energy levels in the cell preventing the ATP-dependent initiation of protein translation^24^; thus, diminishing the growth capacity.

Skeletal muscle requires specific control systems that gradually change with fish age and their physiological (reproductive) stage. The growth hormone/insulin-like growth factor axis is considered the most important endocrine system controlling skeletal growth, although modulation of other hormones like insulin, steroids, etc. has been reported not only to regulate muscle growth and development but also its adaptation to any environmental changes^25^. Many fish species, including yellow perch, exhibit a sexually related dimorphic growth rate. However, this characteristic is usually not apparent in juvenile or young adult perch and sexual dimorphic growth is detected at the onset of gonadal maturation^26^. Shewmon et al.^26^ found that in yellow perch the differences in growth between sexes were coincident with significantly increased levels of testosterone in males and estradiol in females suggesting that sex-dependent growth pattern is a result of an estradiol‐induced increase in feed intake (appetite), feed conversion, and growth of females, together with an androgen‐induced decrease in feed efficiency in perch males^27^. Malison et al.^27^ further argued that the start of dimorphic growth concurred with the onset of gonadogenesis and appeared to be associated with attainment of a certain maturational status, possibly an ontogenetic increase in steroid receptor expression that allowed the fish to demonstrate a growth response. Liang et al.^28^ reported that yellow perch females presented significantly larger size than males when female and male body weights ranged from 65.4 to 89.1 g and from 50.9 to 68.1 g, respectively. The final weight of the fish used in our study ranged from 3.3 to 16.4 g and fish dissection performed at the time of muscle sampling did not identify developed gonads in fast or slow-growing individuals. Hence, the impact of sex and sex hormones on the growth performance that would have been produced at a much later stage, was eliminated from the analyses.

Recently, more focus has also been placed on epigenetic regulation of muscle plasticity and it is evident that DNA methylation events induced by various environmental factors (temperature, nutrition, photoperiod, reproductive hormones, etc.) are closely associated with faster muscle growth^29^. Finally, the MRFs represent another crucial group of molecules that contribute significantly to muscle development including muscle cell maintenance, proliferation, and differentiation, as well as tissue recovery/regeneration after an injury^25^. Fish muscle growth is a complex process, and influenced by a combination of fiber recruitment and the subsequent area increase of these fibers^1^. Depending on both the growth rate and fish species, the contribution of fiber hyperplasia and hypertrophy is different^8,30^. In slow-growing fish species, fiber hypertrophy has a greater effect on muscle growth than fiber hyperplasia^31^. Whereas, fast-growing fish harboring a greater muscle mass largely depend on both the fiber hyperplasia and hypertrophy^30,31^. Although Rescan et al.^32^ reported that compensatory muscle growth, which is considered a period of rapid growth that follows a fasting period, with rates higher compared to previously non-fasted fish, is mediated by stimulation of myofibre hypertrophy. In the current study, fiber CSA of fast-growing yellow perch was more than 1.46 times greater compared to that of slow-growing yellow perch. Moreover, the median CSA of fiber was a greater in the fast-growing group (901 to 1800 μm^2^) compared to the slow-growing group (301 to 1200 μm^2^). Interestingly, CSA distribution of smaller muscle fibers (0 to 900 μm^2^) was higher in fast-growing yellow perch compared to that of median muscle fibers (901 to 1800 μm^2^) (37.8 vs. 32.8%, respectively), and very small fibers with mosaic-like appearance were graphically observed in fast-growing yellow perch. These mosaic-like fibers are considered as newly proliferated and fused fibers from the existing fibers after early juvenile to adult period, known as mosaic hyperplasia^33,34^. Generally, greater growth performance in fast-growing fish is largely associated with increase of total fiber numbers by mosaic hyperplasia compared to slow-growing fish^8^. Thus, in this study, fast-growing yellow perch with a greater body weight seems to be affected by both the muscle fiber hypertrophy and hyperplasia compared to slow-growing yellow perch with a lower body weight.

To confirm the effects of muscle fiber hypertrophy and hyperplasia on growth rate, the expression levels of myoD and myogenin were measured by immunoblotting and image analysis. The sequential expression of the MRFs, including myoD and myogenin, regulates fiber hyperplasia and hypertrophy^35^. MyoD as the primary MRF plays important role in the myogenic proliferation and differentiation which contributes to fiber hyperplastic and hypertrophic growth^36,37^. The secondary MRF myogenin is associated with muscle cell differentiation, and contributes to the postnatal hypertrophic growth^38^. Moreover, Rescan^39^ reported that myogenin protein was visually observed by immunoblotting in mosaic-like muscle fibers in rainbow trout *Oncorhynchus mykiss*. These results similarly were associated with expression levels of myoD and myogenin as in the current study. Fast-growing yellow perch showing mosaic hyperplasia and hypertrophy exhibited greater expression levels of myoD and myogenin compared to slow-growing yellow perch.

## Conclusions

Our results in this study are the first comparison of proteomic profiles and histochemical characteristics between fast- and slow-growing yellow perch. Overall, the results indicate that the faster growth of yellow perch was influenced by both the muscle fiber hyperplasia and hypertrophy, especially mosaic hyperplasia. These hyperplastic and hypertrophic growth were associated with higher expression of not only metabolic enzymes, including GAPDH, glycogen phosphorylase, and aldolase, but also myoD and myogenin as MRFs. Therefore, these proteins could be useful markers of muscle growth to improve the growth performance in yellow perch.

## Supplementary Information

Supplementary Information.

## Data Availability

The original transcriptomic and proteomic data are stored in laboratory notebooks in the MW lab and on university maintained cloud storage systems.
