# Supplementary material for: Proteomic profile and morphological characteristics of skeletal muscle from the fast- and slow-growing yellow perch (Perca flavescens)
Source: Sci Rep. 2021 Aug 11;11:16272. doi: 10.1038/s41598-021-95817-7 (PMC8357941; doi:10.1038/s41598-021-95817-7)

**Proteomic profile and morphological characteristics of skeletal muscle from the fast- and slow-growing yellow perch (*Perca flavescens*)**

Karolina Kwasek^1^,†, Young Min Choi^2^,†, Hanping Wang^3^, Kichoon Lee^4^, John Mark Reddish^5^, and Macdonald Wick^4^*

1 Center for Fisheries, Aquaculture, and Aquatic Sciences, School of Biological Sciences, Southern Illinois University, Carbondale, Illinois, USA

2 Department of Animal Science and Biotechnology, Kyungpook National University, Sangju, South Korea

3 The Ohio State University-South Centers, Piketon, Ohio, USA

4 Department of Animal Scssiences, The Ohio State University, Columbus, Ohio, USA

5 School of Veterinary Medicine, The Ohio State University, Columbus, Ohio, USA

Supplementary files:

**Supplementary Figure 1. Uncropped western blot images of myoD and myogenin in fast- (FG) and slow-growing (SG) yellow perch in Figure 4.**

For western blot analysis of myoD and myogenin, the expression level of β-actin was used as a control. Membranes were cut to minimize antibody use. Predicted molecular weights of MyoD, myogenin, ad β-actin were 35, 34, and 45 kDa, respectively.


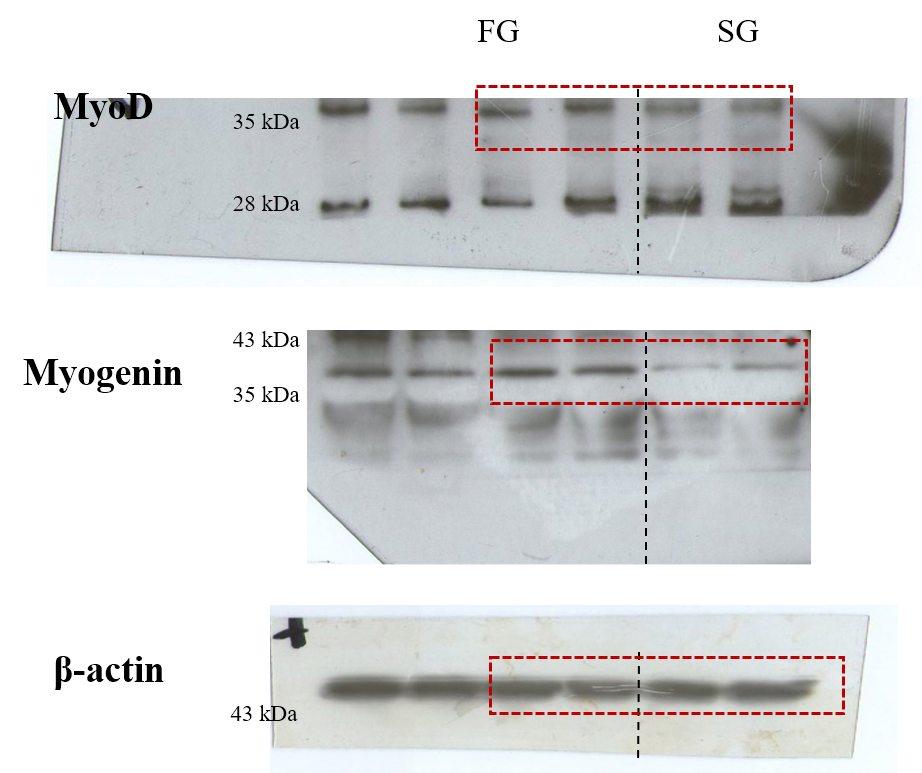


**Supplementary Figure 2. Muscle fibers of yellow perch that were stained using myofibrillar ATPase method after acidic preincubation.**

All muscle fibers were defined as fiber type IIB, and fiber type I was not existed in collected muscle samples of yellow perch. Mosaic hyperplasia of fiber was represented as white arrow.


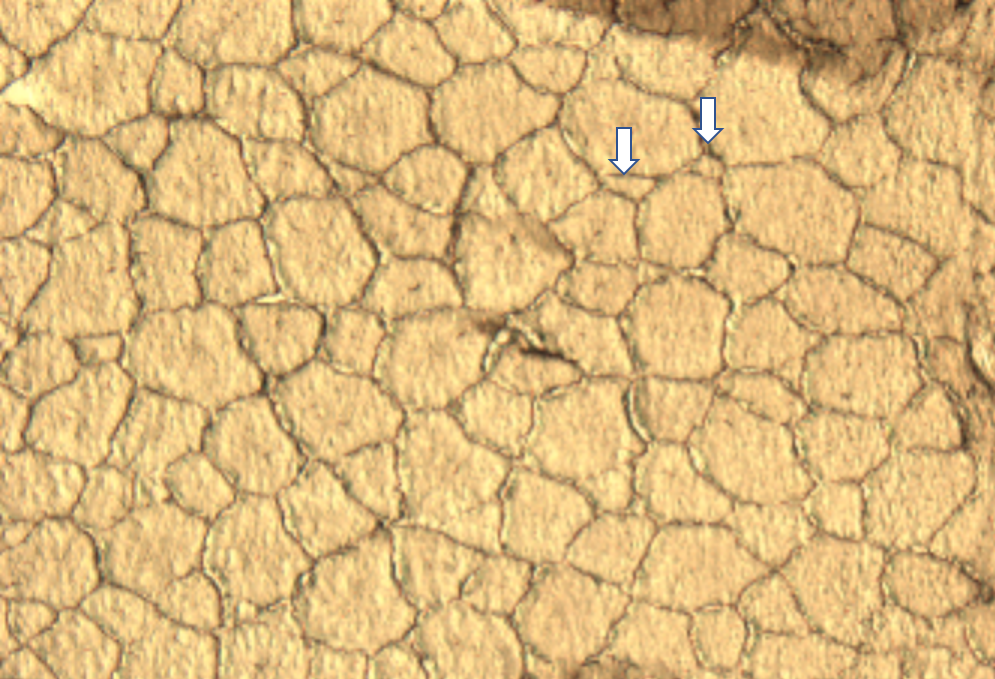


**Supplementary Figure 3. 10% SDS-PAGE of the sarcoplasmic fraction of the muscle of fast- and slow-growing yellow perch.** The bands were analyzed to determine the percentage contribution of each band to the total band area in the lane. Lanes 1, 9, and 15 present marker - the molecular weight standard. The figure presents uncropped gel.


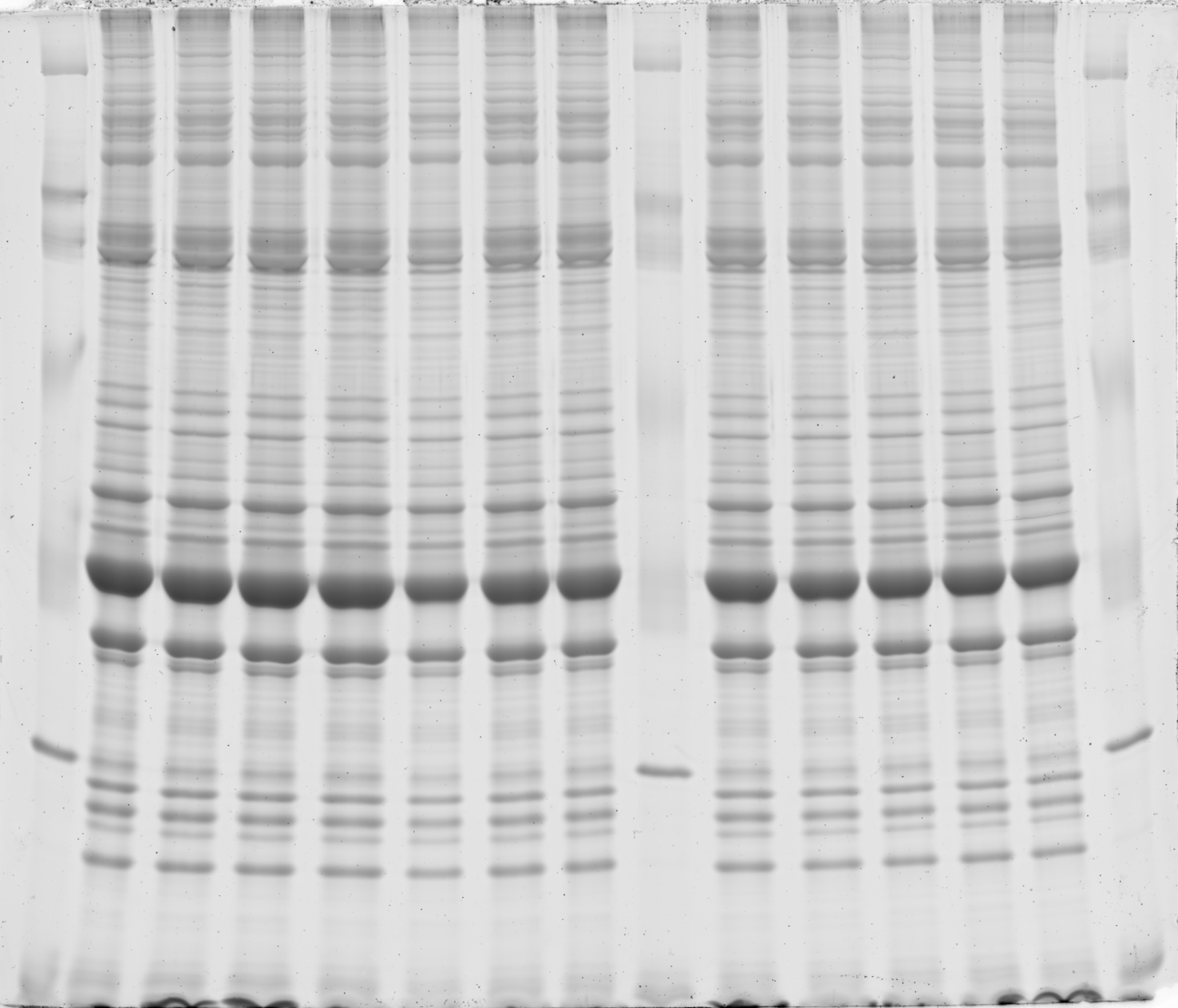

Supplement: Supplementary file 1 — Supplementary Information. [file 41598_2021_95817_MOESM1_ESM.docx]
